# Reporting radiographers and their role in thoracic CT service improvement: managing the pulmonary nodule

**DOI:** 10.1259/bjro.20190018

**Published:** 2020-03-10

**Authors:** Paul Holland, Hazel Spence, Alison Clubley, Chantel Brooks, David Baldwin, Kate Pointon

**Affiliations:** 1 Department of Radiology, Nottingham City Hospital Campus, Nottingham University Hospitals, Nottingham, United Kingdom; 2 Department of Respiratory Medicine, University of Nottingham, Nottingham, United Kingdom

## Abstract

**Objectives:**

The use of cross-sectional imaging in clinical medicine has been a major step forward in the management of many conditions but with that comes the increasing demand on resources and the detection of other potentially significant findings. This, in the context of a shortage of skilled radiologists, means that new ways of working are important. In thoracic CT, pulmonary nodules are a significant challenge because they are so common. Poor and inconsistent management can both cause harm to patients and waste resources so it is important that the latest guidelines are followed. The latter mandate the use of semi-automated volumetry that allows more precise management but is time-consuming.

**Methods:**

Reporting radiographers were iteratively trained in the use of semi-automated volumetry for pulmonary nodules by experienced thoracic radiologists. Once trained in this specific aspect, radiographers completed reporting of pulmonary nodules, checked by radiologists.

**Results:**

Radiographer reporting reduced radiologist time in reporting nodules and measuring their volume. Most of the volumetry was completed prior to the multidisciplinary meeting. This facilitated an increase in the number of patients discussed in 60 min from 15 to 22. Radiographers failed to detect few nodules, although a second read by radiologists is required in any case for other aspects of the reporting.

**Conclusion:**

Reporting radiographers, working with radiologists in a supportive setting, can deliver the radiology in a lung nodule pathway, reducing the time commitment from radiologists and the pulmonary nodule multidisciplinary team members, whilst using this as an opportunity to conduct research.

## Introduction

The 2017 workforce census by the UK Royal College of Radiologists (RCR) found that there has been an estimated 30% increase in overall diagnostic workload in the last 5 years, with increasing complexity of imaging requiring longer reporting time.^1^ RCR estimated there are 1000 fewer consultant radiologists to deliver the demands on the service and that this would increase. The vacancy rate in thoracic radiologists is 7.9% and there has been much use of expensive outsourcing. In Health Education England’s “Cancer Workforce Plan, Phase 1: Delivering the cancer strategy to 2021,” plans include an extra 668 clinical radiologists and an additional 300 reporting radiographers.^2^ In this context, supporting the implementation of new initiatives and evidence-based guidelines is a challenge. The recent expansion of screening for lung cancer is potentially one of the most important ways to reduce lung cancer mortality,^3^ but it has to be done correctly to ensure maximum benefit and cost-effectiveness.^4^ NHS England has published a protocol recommending the use of guidelines in the management of pulmonary nodules^5^ and in the USA there is a detailed implementation guide.^6^ The 2015 British Thoracic Society (BTS) guidelines on the investigation and management of pulmonary nodules were the first to mandate the use of semi-automated volumetry for baseline and follow-up assessment.^7^ Volumetry has since been recommended by the Fleischner Society^8^ as an option, acknowledging the superiority over manual axial measurements.^9^ The most recent version of Lung RADS recommended for use in screening now provides volume thresholds but these are calculated from still uses diameter, assuming nodules are spherical.^10^ Whilst volumetry makes nodule management more efficient in terms of repeat CT, and safer in terms of more accurate risk assessment, it is more time-consuming, especially where it is not a core part of a consultant’s reporting, where there may also be a reduction in sensitivity.^9^

Radiographers have an important role in the NHS in reporting but their role in pulmonary nodules has limited robust evidence. In this context, radiographers have been shown to have lower sensitivity and higher overall rates than radiologists overall, but there is some overlap.^11^ ^12^ In the United Kingdom Screening Trial (UKLS) the mean (SD) sensitivity of the four radiographers was 71.6% (8.5) compared with 83.3% (8.1) for the three radiologists. Radiographers achieved lower sensitivity and detected more FPs per case than radiologists in 7/10 and 8/10 radiographer-radiologist combinations. In 3/10 and 2/10 combinations, there was no difference in sensitivity and FPs per case between radiographers and radiologists. For nodules ≥ 100 mm^3^ in volume or ≥5 mm in maximum diameter, radiographers achieved lower sensitivities than radiologists in only 5/10 radiographer-radiologist combinations (range of difference 16.1–30.6%; *p* < 0.05) and not significantly different in the remaining 5/10 combinations.

Again, in UKLS, radiographers were shown to be effective as concurrent readers.^13^ The use of computer-aided detection is a potential way to reduce reporting time and increase accuracy but is not yet widely used in nodule management. This is the subject of ongoing research.

The primary aim of this service change project was to train radiographers to report all aspects of pulmonary nodules, including volumetry, on thoracic CT. Secondary aims were to support the functioning of the pulmonary nodule multidisciplinary team (MDT) meeting and encourage research.

## Methods

The publication of the BTS guideline in July 2015 prompted the formation of a virtual nodule service and the radiological standard adopted for nodules was volumetry. The impact on thoracic radiologist time was anticipated to be significant and so a business case for radiographer reporting was written based on potential reduction of radiologist reporting time.

Prior to formulating a role for the radiographer to report pulmonary nodules other potential solutions were explored. Initially lectures were delivered to radiologists and registrars on how to access and use the volumetry software, uptake of which was limited. The volumetry software has subtleties and idiosyncrasies which lends itself to users who use it regularly. It was therefore felt that this role was best-suited to a dedicated group of individuals, familiar with the system, processes and guidelines to ensure best practice and governance with reports and the MDT setting. The increase in outsourced reports resulted in many cases where volumetry was not performed due to remote reporters not having access to the volumetry software.

The use of the software is time-consuming and technical, training radiographers was a logical step over training registrars, as radiographers are not on training rotations to other centres. The non-thoracic consultant radiologists are already working with many different analysis packages in their specialist areas. The radiographers and thoracic radiologists offered to support colleagues wanting to become proficient, but the reality has been that the cases are now referred to the nodule MDT service.

It was anticipated that the radiographers would reduce the amount of time it took for the radiologists verify the report so that they did not have to attempt the volumetry and that furthermore this would be included in a structured report (Supplementary Material 1).

The radiographers had a background of CT training between 3 and 15 years’ experience, they either had PGCert qualification in CT imaging, CT virtual colonography reporting and chest and abdomen X-ray reporting (due to complete Summer 2019). The radiologists were all thoracic specialists with between 3 and 20 years’ consultant grade experience.

Risk assessments were carried out on the existing arrangements and the proposed new role. Pre-existing risks were highlighted as:

- Insufficient radiologist time allocated to allow for a robust reporting service for this patient group.
- The CT scanning protocol for pulmonary nodules was not standardised across the Hospital’s CT sites.
- No standardised report template for the follow up of lung nodules.
- Reports did not consistently provide the required volumetric data or volume doubling times (VDT).
- Follow-up scan advice was not always in keeping with BTS pulmonary nodule guidance.
- There was no in-house capacity to train other users to use the nodule analysis software.

Table 1 shows an assessment of the strengths, weaknesses, opportunities and threats analysis.

**Table 1.** SWOT analysis

Strengths	Weaknesses
▪ Lung nodules are managed on a pathway, via a virtual clinic. ▪ Clearly defined timing of interval scans. ▪ Structured report format ensuring consistency. ▪ Standardised imaging protocols. ▪ Improved patient communication and experience.	▪ Potential decrease specialist registrar training opportunities. ▪ Reduced radiographer clinical time.
Opportunities	Threats
▪ Supports recruitment and retention. ▪ Improved multidisciplinary team (MDT) working. ▪ Radiographer vetting.	▪ Risks of further pathology being overlooked.

### Training

Three radiographers were trained according to an apprentice system, they equated to a total of 3 days (1 day each) reporting and MDT preparation per week. This involved working with thoracic radiologists and each other to observe reporting and compose reports. The reporting radiographers were recruited solely to report pulmonary nodule follow-up cases once nodules had been detected. Their training was based on exposure to follow-up nodule cases only during the teaching sessions.

Radiographers are not part of the CT reporting system that generates new referrals to the nodule service but, later, added to their role, reporting volumes of nodules on these scans prior to the nodule MDTM(Multi-disciplinary team meeting). The radiographer, through one-to-one radiologist sessions of dedicated nodule reporting and self-guided study, was expected to recognise the types of nodules, detect new nodules and refer to BTS guidance for morphology and recommended follow up.

The radiographers had a dedicated RIS(Radiology Information System) reporting group code to enable them to identify cases to report. The booking team would allocate these patients to the “pulmonary nodule” reporting group on the RIS system depending on the specific authorisation protocol identified on RIS.

The radiographers had some application training on the volumetry platforms that were available.

The nodule volumetry software was GE Volume Viewer v.12.3—Lung VCAR, (USA version) and Siemens Syngo Via VB20A MM Oncology, (EEC version). The Lung VCAR was included in an oncology module within the local GE PACS and Syngo Via as a thin client-based platform. This meant that all previous images could also be accessed to check for resolution or growth and to calculate VDT where appropriate. The same platform was always used for comparative volumetry measurements.

The reporting style and structure evolved over time depending on the radiographer’s experience and confidence gained throughout their training.

The radiographers would do comparative volumetry over the past interval (usually at three or 12-month follow-up), if there were new nodules seen, the radiographer would attempt volumetry and include this in the final report and conclusion and recommendations accordingly. If this was missed, the discrepancy would be fed back to the radiographer.

After approximately 6 months of these sessions and attendance at the MDT, the radiographer would start primary reporting. Feedback from the thoracic radiologist verifying the report was done in face-to-face or more informal email depending on the discrepancy.

Prior to the initiation of the project, a structured reporting form was developed (Supplementary Material 1).

Primary reports were generated itemising each nodule of concern, including location, shape and density of nodule, ensuring comparison with prior studies. Close to 95% of all reports included semi-automated volumetry measurements unless segmentation was judged to be unreliable or was not possible due to CT acquisition. Conclusions always include a recommendation on follow-up interval (if applicable). The accuracy of the automated volumetry segmentation and report content, including any new nodules, is verified by a radiologist, having first been checked by the radiographer. Any discrepancies and alterations to the report are documented by the reporting radiographers for every case to highlight learning themes and to enable changes to practice.

The pathway is supported by an MDT meeting which involves thoracic radiologists, chest physicians and reporting radiographers. The MDT provided an opportunity for further checks on the radiographer report.

Primary reports were and are always verified by a thoracic radiologist, there is currently no mandate to allow reporting radiographers to verify their reports independently.

The time for training to be judged complete was 6 months, although there is continual checking of reports by the radiologists, formal one to one and informal feedback. After a further 6 months or around 50 cases, a competency assessment was completed by the Lead Thoracic Radiologist as part of the “Working in New Ways” package, they are signed off to provide first reports and re-numerated appropriately.

An existing Nottingham University Hospitals “*Working in New Ways”* package was utilised to define and contextualise the role of the pulmonary nodule reporting radiographer, clearly outlining responsibilities, training and required competencies.

This provided a framework for:

- Examination preparation
- Generating reports
- Providing management advice specific to the pulmonary nodule follow-up pathway
- Attendance and input to MDT meetings.

The textures of the nodules are reported using BTS terminology, which not all non-thoracic radiologists will be familiar with. This then improves the risk assessment for malignancy and confidence in the appearances of typical, atypical and non-typical peri-fissural nodules.^13^

## Results

There were around 30 nodules per week requiring analysis on the pathway and 25 patients discussed at the weekly nodule MDT meeting. A total of 2312 people were discussed between^8^ May 2017 and July 2019. All CTs, both baseline and follow-up that were referred to the nodule service were viewed and discussed at the nodule MDT. Virtually no follow-up nodules were reported by non-thoracic radiologists.

### Quality assurance measures

The radiographers, working with thoracic radiologists, developed a standard operating procedure based on the BTS guidelines for nodule follow-up. Thus, radiographers were able to justify and protocol the request for the scan, making the pathway almost entirely radiographer led. The components developed were:

Template report for the radiographers to work towards.

Inclusion criteria for reporting.

Guidelines for what size and type of nodules to report and its relevance to BTS guidance.

All CTs were checked by experienced radiologists as well as being discussed at the nodule MDT meeting. A database is held of all cases that have been reported. A record is kept of any clinical interpretation discrepancies, any changes to clinical advice and any grammatical changes.

Radiographers were with additional support and training encouraged to include more detail within their reports over time so latterly there has been more detail in the report to be changed. This has therefore probably increased the likelihood that reports (and not the volumetry figures) may have been altered as the radiographers were providing more detailed content.

### Quality control

There were three reporting radiographers of varying levels of experience audited, one started in the May 2017, while another joined in March 2018 and the third in June 2018. In the first month, the volumetry was not altered in 68% of cases and the number of reports changed was 82% (including all aspects of the findings), in the last month the volumetry accuracy was not altered in 93% of cases and 44% of reports were changed.

Overall, a total of 732 CTs had been reviewed by radiographers between October 2017 and July 2019. In the first 6 months (first trained radiographer), 3.5% (3 out of total 85 cases) of nodule volumes/interpretation of nodules were changed by radiologists. In the last 6 months, where three radiographers were reporting cases separately, 3% (13 out of total 420 cases) of the nodule element of reports were changed. The discrepancies between radiographers and thoracic radiologists followed a common theme where radiographers were more cautious, were more inclined to continue interval follow-up imaging rather than discharge a patient. In the last 12 months, the follow-up recommendations between radiographer and radiologist/MDTM agreed in 92% of cases.

Radiographer reporting time ranged from 15 to 60 min, reflecting experience and the total time from accesing the image to final report, in the last 6 months the reporting times have shown consistancy between the radiographers(Table 2). Radiologist confirmation time per scan was 7–10 min. The time for radiologists to perform volumetry per case and check for nodules was on average 23 min without radiographer reporting and an average of 9 min with, a saving of 14 min. Thus, the total radiologist reporting time saved, assuming 1200 reports per year is around 5.38 h per week (52 weeks covered per year).

**Table 2.** First and last 6 months results of the radiographer reporting

	Radiographer 1	Radiographer 2	Radiographer 3
Reporting time (average on reports in the last month)	20 min	23 min	25 min
Nodule volume not altered First 6 months	95%	74%	80%
Nodule volume not altered Last 6 months	100%	93%	97%

The radiographer may recommend further imaging if the nodule is suspicious or has a borderline VDT, this may be over-ruled in the MDTM.

In the last 18 months, no radiographer reports led to an incorrect management decision.

### Additional roles

Training led to the following additional roles and responsibilities:

- Ability to justify and protocol requests for nodule follow-up.
- Scanning, reporting and recommending follow-up interval.
- Details of audit, CPD, record of MDT attendance.
- Standardised imaging parameters.
- Standardised measurement of volumetry.

### Service impact

1. The thoracic radiologists have widely reported that verifying the primary reports compared to them exclusively reporting was done in half the time.
2. Since radiographer reporting of pulmonary nodules has been implemented virtually, all new patients and all of the follow-up patients have had volumetry or attempted volumetry (where not possible). This has led to prompt decisions and the number of patients discussed per week rising from 15 to 25.
3. The current workload of pulmonary nodule reports is approximately 1000–1200 of follow-up cases per year, this will include addenda that the reporting radiographer will add to new incidental cases in preparation for the MDT.
4. Radiographer-led nodule reporting has led to the closer standardisation of nodule imaging within the hospital sites. Radiographers discovered that the volumetry software required certain acquisition slice thickness and algorithms not previously remedied to improve volumetry accuracy and segmentation.
5. The reporting radiographers are familiar with the strengths and limitations of the in-house software to provide accurate volumes for nodules, quoted in the reports, enabling the MDT to efficiently discharge benign nodules.
6. The radiographers will turnaround reports largely within 3–5 days; any progressing nodules are alerted to the thoracic radiologist team for urgent verification to enable prompt MDT review.
7. All nodule reports were originally allocated to the thoracic radiologists for reporting, these are now allocated to “Nodule” reporting to enable the radiographers to effectively find cases to report.
8. The radiographers have become actively involved in research developing a new artificial intelligence (AI) system for nodule risk assessment (National Institute for Health Research II-LB-0716–20006).

## Cost considerations

The time for training of radiographers was minimal as the radiographers attended the reporting sessions of the radiologists and assisted with some aspects relating to nodule volumetry. Thus, time was saved for the radiologist “in return” for time in teaching. Overall this was thought to be neutral with no appreciable reduction in radiologist reporting speed. Radiographers used 7.5 h per week for 6 months before they began independently reporting nodules. Our average estimate of 14 min radiologist time saved per report allows an approximate estimate of the balance of costs for an implemented service below:

## UK costs

### Radiologists

14 mins × 1200 reports = 280 h per year = 5.4 h per week. Cost saving = 5.4/40 × £9,1250 (average consultant salary) = ~£1,2300 per annum.

In addition, the MDT preparation would account for around 1.5 h per week.

90 mins x £9,1250 (average) = £3420.

Total amount saved per annum = £1,5720.

### Radiographers

Average radiographer reporting time is 23 min × 1200 = 8.8 h per week. Additional cost = 8.8/40 × 3,7570 (band 7, mid-point radiographer salary) = £8265.

### Balance of costs

Net cost = 8265−15720 = −£7455 (saving)

## Discussion

The role of a reporting radiographer for appendicular skeletal plain films is well established within radiology. The impact has been shown to reduce patient waiting times, improve safety and decrease costs.^14^ The role of the chest X-ray reporting radiographer has been slowly evolving, with the College of Radiographers accrediting a postgraduate certificate in 2002.^15^ Radiographer reporting has increased reporting capacity where introduced^16^ and has been shown to be equivalent to radiologist reporting in some settings.^17,18^ It was therefore a natural step, alongside radiographer reporting of plain radiographs, to look at the feasibility of radiographer reporting of thoracic CT as part of a pathway. This, along with limited radiologist reporting time, the increased demand for pulmonary nodule management according to new guidelines, ever-increasing incidental nodule detection and the phased introduction of CT screening for lung cancer drove our project forward. A sound argument was made in the business case and after 6 months of iterative apprentice-style training radiographer reporting was integrated into the pathway. Our study shows how this can be done and the positive impact that is derived after a relatively short period of time. The role has expanded the number of reporters for pulmonary nodule follow-up at a time when the service was struggling to maintain report turnaround times, while ensuring that pulmonary nodules reports include a specific data-set including volumetry.

The role has supported optimal protocolled adherence to the BTS recommendations by ensuring optimal methods (volumetry) were used for baseline and growth assessment. Radiographers have acquired a greater understanding of the scanning protocols and of the volumetry software and the outputs.

This has supported the pulmonary nodule pathway, with timely standardised reports that provide the volumes of nodules and comments on the quality of nodule segmentation facilitate recording of data for the MDT, audit and research. Imaging protocols for pulmonary nodule follow-up is now standardised with scanning protocols (slice thickness and algorithms have now been optimised for the volumetry platforms to enable reliable segmentation) consistent across two hospital sites.

This has supported the pulmonary nodule pathway, 3–5-day report turnaround by the radiographers that provide the volumes of nodules, in turn facilitated by a standardised report that includes nodule volumes and comments on the quality of nodule segmentation. Furthermore, the radiographers alerting the radiologists to progressing nodules has better fast-track cases to the MDT, resulting in quicker clinic appointments and ultimately prompt treatment.

Our system of reporting by radiographers followed by radiologist review not only reduces radiologist reporting time but provides quality control, inevitably a concern when modifying traditional approaches. The MDT discussion provides a further check. It is accepted that the radiographer reporting element here is only one element of the thoracic CT but this time-consuming element can arguably be done better by radiographers who may be rather better at following protocols and have more protected time allocated to this activity.

### Future developments

The use of AI in radiology is a fast-developing field and there are several nodule detection, risk prediction and management systems currently in development that show promise,^19–22^ including one that the authors are currently developing and testing.^23^ This offers the potential to further enhance the role of clinicians, including radiographers and inevitably there will be a need for validation of AI outputs which may be time-consuming.

As radiographers scan their own patients, there is the opportunity to explain the potential outcome from the scan to patients, keeping them informed in a timelier manner than is currently seen. Patients are often anxious about their scan results and this can provide early reassurance for the majority and rapid triage for the minority who need further work-up. The developing CT screening programmes may also benefit from a more active approach from radiographers in managing the outcome of the CTs. Immediate radiographer triage after an immediate CXR report may have a marked effect in reducing the time to diagnosis,^24^ so this may also apply to CT.

## Conclusion

The combination of robust training packages, accurate documentation and the expertise within the thoracic radiologist and pulmonary nodule team has provided broad teaching and support for the reporting radiographer’s development. This has reduced radiologist reporting time, allowed optimal guideline adherence, improved reporting and facilitated data acquisition. The radiographers report will almost always provide volumetry measurements due to their continually expertise using the software. Although difficult to quantify, this has undoubtedly provided more decisive outcomes, enabling early discharge and reduced anxiety for patients with benign nodules while optimally identifying definitive progression to enable faster treatment. This has greatly assisted the management within the MDT, allowing more patients to be discussed in the allotted time. These improvements were achieved without influence on clinical decisions or safety.

Although there has a cost implication for introducing this service, the benefits are far reaching, better patient outcomes, frees up CT demand by early discharge, faster verification process, better prepared MDTs, better understanding of the volumetry software and its limitations and more time for thoracic radiologists to perform more complex work within their job plans such as cardiac CT and MRI, CT-guided biopsies and other interventional procedures.

The reporting radiographers have become our “superusers” in this area, and now provide training and support for consultants and specialist trainees looking to further develop skills and understanding of pulmonary nodule reporting and pathways.

We now plan to explore the real-life implementation of radiographer and radiologist reporting in the context of the imminent NHSE (NHS England) Lung Health Check programmes.^5^
